# The effect of smartphones on the self-rated health levels of the elderly

**DOI:** 10.1186/s12889-022-12952-0

**Published:** 2022-03-15

**Authors:** Xian Liang, Feixue Xiong, Fangting Xie

**Affiliations:** 1 College of Economics and Management, Zhejiang A&F University, 311300 Hangzhou, China; 2 School of Agronomy Sciences, Jiangxi Agricultural University, 330045 Nanchang, China; 3 School of Economics and Management, Jiangxi Agricultural University, 330045 Nanchang, China

**Keywords:** Self-rated health level of the elderly, Smartphone usage, Smartphone usage ability, Smartphone usage purpose

## Abstract

**Background:**

Due to the penetration of Internet use and the popularity of “Internet + elderly care” among seniors in recent years, the elderly are gradually integrating into the information society. This study examined the impact of smartphones on the self-rated health levels of the elderly.

**Methods:**

We studied 3042 elderly people over 55 years of age in Jiangxi, China in 2018. The effect of smartphones was measured from three aspects: smartphone usage, smartphone usage ability, and smartphone usage purpose, and the multivariate ordered logistic model was applied. Furthermore, considering the potential endogeneity of the smartphone usage of elderly people, the propensity score matching (PSM) method was used to analyze the net effect of smartphones on the health levels of the elderly.

**Results:**

(1) The use of smartphones had a significant positive impact on the self-rated health levels of the elderly, with its significance being at the level of 1%. Smartphone usage ability, and using smartphone to learn or search for health information, had significant positive impacts (at the level of 5%) on the self-rated health levels of the elderly. (2) The k-nearest neighbor matching, kernel matching and radius matching methods were used to calculate the net effect of smartphone usage on the self-rated health levels of the elderly. The results were 13.26, 15.33 and 14.80%, respectively. (3) The age of the participants significantly (at the level of 1%) negatively affected their self-rated health levels. Other characteristics of the elderly, including income, education level, living with children or spouse and children’s living conditions, significantly (all at the level of 1%) positively affected their self-rated health levels.

**Conclusions:**

Smartphone usage, smartphone usage ability, and smartphone usage purposes all improved the self-rated health of the elderly. The Internet factor should be focused on in the process of active aging. We should improve the Internet use ability of the elderly through voluntary training or public lectures.

## Background

In recent years, the Internet, and especially smartphones, have enriched the intergenerational interaction mode between the elderly and their children, and enriched the social network and recreational activities of the elderly [1]. The China Internet Network Information Center (CNNIC) released the 47th Statistical Report on Internet Development in China on February 3, 2021. According to the report, the number of elderly netizens in China increased from 5.8% in 2010 to 26.3% in 2020 (CNNIC, 2021). The use of smartphones permeates every aspect of the lives of the elderly. More empirical studies from different perspectives are needed to provide evidence to deepen the understanding of the relationship between smartphones and the health of the elderly.

The rapid development of smartphones has not only impacted the lifestyle of young people, but also changed the lifestyle of the elderly. With the continuous development and progress of Internet technology, smartphones now intelligently combine different fields, such as education, medical care, transportation, and finance. Internet smart pension also provides a feasible idea for aging policy [2]. With the help of smartphones, many online health services and communication platforms have gradually emerged in many countries in recent years, which is also considered as an important development trend for the future elderly health industry [3]. The elderly can obtain knowledge about disease prevention, rehabilitation care and drug use instructions through online search tools. Smartphones have enhanced the health information literacy (HIL) capabilities of the elderly [4].

### The present study

In previous literature, studies on the self-rated health of the elderly have mainly focused on the analysis of the factors influencing their self-rated health. Compared with other age groups, the proportion of Chinese elderly smartphone users has always been very low, and the large-scale integration of the elderly into the information society only begun relatively recently. In recent years, researchers have conducted numerous studies on the impact of smartphone usage on the elderly’s self-rated health [5].

#### Analysis of factors affecting the health of the elderly

In the analysis of factors affecting the health of the elderly, scholars’ research mainly focuses on the analysis of subjective and objective factors. Among the subjective factors, the age, education level, and marital status of the elderly has been found to have a significant impact on the health of the elderly [6].

Some scholars have pointed out that ageing leads to the degradation of the elderly and reduces their ability to live socially [7]. The higher the education level, the more health knowledge the elderly can acquire, which is conducive to the improvement of their health status [8]. Married elderly people may have better mental status [9]. Studies have further pointed out that divorce and remarriage provide health protection for the elderly, especially for elderly females [10]. Good lifestyle habits also improve the health status of the elderly, such as regular exercise and reducing smoking and alcohol habits [11]. Leisure activities as a major part of their lives also affects the elderly’s health. For example, participation in social activities and light outdoor activities improves the health of the elderly [12].

Among the objective factors, occupation, intergenerational support and social support all affect the health levels of the elderly. In terms of occupation, elderly people engaged in mental work will have better health. The possible reason is that they often have a higher social status and accordingly receive more health resources [5].

In terms of intergenerational support, living with family members also raises the health levels of the elderly. The care and companionship of children and spouses has been found to have a positive impact on the psychological states and social integration of the elderly [13].

In terms of social support, some scholars have found that social support has a significant positive impact on the self-rated health of elderly people of all ages [1]. The more society publicizes the virtue of respecting and caring for the elderly, the more elderly people are motivated to interact with others and seek help [14].

In terms of medical policies, Zhao [15] used the data from the CFPS survey database in 2018, confirmed that the total medical consumption, or out-of-pocket medical expenses of the elderly has a negative impact on their health.

#### Analysis of the impact of internet use on the health of the elderly

At present, academic research on the health level of the elderly mainly focuses on the subjective and objective factors. Less attention has been paid to the integration of older adults into the information society and the social problems brought about by the “digital divide”. Among the existing studies, most scholars hold positive opinions about the impact of smartphones on elderly users’ health. The use of smartphones has enriched the entertainment activities and leisure lives of the elderly [16].

However, the existing research mainly focuses on the smartphone usage of the elderly, and few studies have examined the specific pathways by which smartphone usage affects their health [17]. Scholars have different views on the nature of smartphones’ influence on the health levels of the elderly. For example, some scholars have pointed out that the learning function and frequency of smartphones can act as an intermediate pathway that affects the health level of the elderly. Studies have found that health awareness is higher among older adults who use smartphones frequently, especially those who search for health information frequently [18].

The Internet affects the mental health of the elderly differently than it affects physical health [19]. Smartphone usage helps elderly people who are lonely or intellectually disabled to access nursing guidance and perform mental healing [20]. Some scholars argued that the use of smartphones increases the loneliness of the elderly, negatively affecting their health [21]. Zhao [15] further pointed out that smartphone has a greater impact on the mental health of the elderly users than it does on their physical health, and this impact increases over time [22]. Zhu [23] raised different issues after her empirical analysis of elderly people of different ages. She suggested that smartphone has a greater impact on the psychological self-rated health of the middle and lower aged users compared to the older ones.

With the rapid development of the Internet, the impact of smartphones on the health levels of the elderly cannot be ignored [24]. The relationship between the Internet and the health level of older adults can be meaningfully explored by analyzing the smartphone usage, smartphone usage ability, and smartphone usage purposes of the elderly [25]. However, the existing research lacks an in-depth analysis of the relationship between smartphones and the health levels of the elderly. Among various types of devices used to access the Internet, smartphones are the most accessible one in the daily lives of the elderly. Therefore, this paper chooses to focus on smartphones in order to explore the relationship between Internet and the health of the elderly.

The innovations of this paper are: First, in recent years, the use of smartphones has gradually penetrated into the higher age group, which has brought to active ageing and the construction of intelligent old-age systems a new research perspective. Second, there are few studies on the relationship between smartphone usage and the self-rated health levels of the elderly. Further, little consideration is given to the influence of smartphone usage ability and smartphone usage purposes on these self-rated health levels. Research on the impact mechanism between smartphone use and the self-rated health levels of the elderly can provide policy suggestions for intelligent elderly care.

### Theoretical assumptions

The use of smartphones helps the elderly to bridge the digital divide and access the dividend express of the information society, which promotes social harmony [26]. It also reduces the barriers for the elderly in their daily lives, such as travel, medical treatment, and quick payment [27]. The use of the Internet helps the elderly integrate into the “digital age” [28]. It also effectively alleviates the psychological gap caused by the difficulty of adapting to the transition from a “working state” to a “leisure state” [12]. Accordingly, this paper makes the following assumption:

- H1: The use of smartphones can improve the self-rated health of the elderly.

Meanwhile, having a strong ability to use smartphones means that an elderly person can achieve convenient functions such as learning, medical treatment, and shopping, which are considered to be beneficial to their physical and mental health [29]. Accordingly, this paper makes the following assumption:

- H2: The stronger the ability to use smartphones, the higher the self-rated health levels of the elderly.

The development of “Internet + medical health” provides the elderly with health management services such as online medical consultation, medication guidance, management records, and health knowledge [30]. Wu [22] pointed out that 73.68% of the elderly tend to live at home. It is necessary to promote the combination of smart medical care and elderly health care, such as monitoring the health data of the elderly and efficient medical treatment [31]. The traditional medical treatment method is no longer suitable for the medical needs of modern elderly people [32]. More and more elderly people are learning and acquiring health information on the Internet to enrich their knowledge of medical protection and improve their health literacy [33]. Accordingly, this paper makes the following assumption:

- H3: The use of smartphones to study or obtain health information can improve the self-rated health of the elderly.

## Methods

### Data source

Data were collected in 2018 for a questionnaire survey on the application of network technology for elderly people over 55 in Jiangxi Province. After the questionnaire surveys and interviews, data analysis was conducted by descriptive statistical analysis and the card square test. The content of the questionnaire includes: (1) demographic data of the elderly (including age, gender, education level, family status, health, work status, living status, etc.); (2) the network situation of the elderly (including smartphones, computers, Internet usage, etc.); (3) the needs and attitudes of the elderly towards network technology. A total of 4236 questionnaire responses were obtained, including 3042 valid questionnaire responses related to the study topic.

### Variable selection

The dependent variable in this paper was “self-rated health levels”. According to the five categories that respondents used for their answers—“very poor, poor, average, good, very good”—dependent variable values were assigned from 1 to 5. The core independent variables were “Whether the elderly use smartphones”; “The ability of the elderly to use smartphones”; “Whether to use smartphones to search for learning and health information”.

The control variables in this paper were selected from three aspects: individual characteristics, family characteristics, and socio-economic characteristics. For individual characteristics, the age and education level of an elderly person have been found to affect their health and thinking ability [34]. The study thus chose “gender”, “age”, and “education level”.

The second is family characteristics. The family is the center of life of the elderly in their later years, and it is the main material source and spiritual sustenance of most elderly people [35]. The study therefore chose “children’s living conditions”, “number of children”, and “living conditions”.

The third is socio-economic characteristics. The residence and income of an elderly person has been found to affect their medical security and living conditions [36]. The research thus chose “residence”, “income”, and “current job”. The definition and description of the variables are shown in Table 1.

**Table 1:** Variable description

Variables	Definition	Minimum	Maximum	Mean	SD
*Dependent variable*					
*Health*	Self-rated health level; 1 = very poor; 2 = poor; 3 = average; 4 = good; 5 = very good	1	5	3.27	0.80
*Independent variables*					
*Usage*	Whether use smartphone; 1 = yes; 0 = no	0	1	0.34	0.47
*Ability*	Smartphone usage ability; 1 = very poor; 2 = poor; 3 = average; 4 = strong; 5 = very strong	1	5	4.07	1.45
*Purpose*	Whether use smart phone to search for learning and health information; 1 = yes; 0 = no	0	1	0.61	0.48
*Control variables*					
*Gender*	Gender of elderly; 1 = male; 0 = female	0	1	0.68	0.46
*Age*	Age of elderly; 1 = 55-60 years old; 2 = 61-65 years old; 3 = 66-70 years old; 4 = 71-75 years old; 5 = 76-80 years old; 6 = over 80 years old	1	6	3.11	1.34
*Edu*	Education level of elderly; 1 = primary school or below; 2 = junior high school; 3 = senior high school/technical secondary school; 4 = University; 5 = master degree or above	1	5	3.19	0.80
*Living situation*					
*Spouse*	Living with spouse; 1 = yes; 0 = no	0	1	0.54	0.49
*Children*	Living with children; 1 = yes; 0 = no	0	1	0.14	0.35
*SC*	Living with spouse and children; 1 = yes; 0 = no	0	1	0.25	0.43
*Conditions*	Children’s living conditions; 1 = poor; 2 = average; 3 = good; 4 = very good	1	4	2.69	0.68
*Number*	Number of children; 1 = 0; 2 = 1-2; 3 = 3-5; 4 = 6 and over	1	4	2.42	0.53
*Income*	Elderly’s monthly income; 1 = 1000 yuan and below; 2 = 1000-2000 yuan; 3 = 2000-3000 yuan; 4 = 3000-4000 yuan; 5 = 4000 yuan and over	1	5	3.45	0.90
*Current work*					
*Retirement*	1 = yes; 0 = no	0	1	0.83	0.37
*Emp*	Employed after retirement; 1 = yes; 0 = no	0	1	0.11	0.31
*Business*	Own business; 1 = yes; 0 = no	0	1	0.02	0.14
*Residence*	Place of residence; 1 = rural; 2 = towns; 3 = counties, cities, and districts	1	3	2.94	0.28
Number of samples	3042				

### Model selection

This paper explores the impact of Internet factors on the self-rated health of the elderly. The study was conducted in two steps. The first step, the multivariate ordered logistic model, was selected to explore the impact of elderly smartphone usage, smartphone usage ability, and smartphone usage purposes on the self-rated health of the elderly. The study divided self-rated health levels, from weak to strong, into five types. At this time, the dependent variable was a degree variable, and the logistic model was used to estimate the influencing factors of the self-rated health of the elderly. The setup model was as follows:

1 $${Y}_i={\beta}_{0i}+{\beta}_{1i}{X}_1+{\beta}_{2i}{X}_2+{\beta}_{3i}{X}_3+{\beta}_{4i}{X}_4+{\mu}_i$$

In formula (1), i = 0 or 1, Yi indicates the elderly self-rated health level, β indicate the coefficient vector group, and μ indicates the random error term. Next, the robustness of the model was verified. In the econometric analysis, robust standard errors were used to eliminate the influence of heteroscedasticity on the model results. The above econometric models were all implemented by Stata 16.0.

The second step, the propensity score matching (PSM) method, was selected to measure the net effect of smartphones on the health levels of the elderly. Since the use of smartphones by the elderly is determined by their situation rather than random decisions, there may be selective deviations between the use of smartphones and the elderly’s self-rated health levels. In addition, the individual characteristics of the elderly would also have an impact. To control the possible selection bias, this paper used the propensity score matching (PSM) method to eliminate the endogenous problem. It also used the PSM method to measure the net effect of the use of smartphones on the self-rated health levels of the elderly [1]. The model set the elderly people who use smartphones as the control group, and the elderly who did not use smartphones as the experimental group, realizing the randomization of non-random data. At the same time, the model controlled the similarities of the individuals in the two groups in various dimensions to the greatest extent, and carried out the effects of the two groups. By comparison, the net effect of the use of smart phones on the self-rated health levels of the elderly was obtained. The specific model is:

2 $${y}_i={y}_{0i}+\left({y}_{1i}-{y}_{0i}\right){D}_i$$

In formula (2), Di is the processing variable. When i is equal to 1, it means that the individual is in the experimental group. When i is equal to 0, it means that the individual is in the control group. In this paper, the elderly people who use smartphones were the experimental group, and the elderly people who do not use smartphones were the control group.

3 $$ATT=E\left({y}_1\left|\right.D=1\right)-\Big({y}_0\left|D=1\Big)\right.$$

In formula (3), ATT represents the average processing effect. That is, the net effect of smartphone use on the self-rated health levels of the elderly is obtained through the difference between the experimental group and the control group. Y1 indicates that the self-rated health levels of the elderly are affected by smartphones, and Y0 indicates that the self-rated health levels of the elderly are not affected by smartphones. When D is equal to 1, it indicates that the elderly are in the state of using smartphones.

This paper used three methods to match the experimental group and the control group. These three methods were k-nearest neighbor matching, kernel matching and radius matching in the caliper, respectively; psmatch2 was used in stata16. The three matching methods are briefly described as follows:

1. k-nearest neighbor matching in the caliper: Based on the propensity score values for individuals in the experimental group, k different and similar values in the control group for matching are found. In this paper, k was set to 1, for one-to-one matching.
2. Kernel matching: By setting the propensity score bandwidth, all control group individuals matched with the experimental group are weighted. The closer the distance to the individual, the higher the weight. The kernel function and bandwidth in this paper used default values for the kernel matching.
3. Radius matching: By setting the radius in advance, searching for all control samples within the set radius and performing weighting processing, a virtual sample that can be matched with the current sample is obtained. In this paper, the radius is set to 0.05 in the radius matching.

## Results

### Descriptive statistics

The mean self-rated health level of the respondents in the sample was 3.27. The elderly with “very poor” health levels accounted for 1.07%, those with “poor” health levels accounted for 12.31%, those with “average” health levels accounted for 51.82%, those with “good” health levels accounted for 27.61%, and those with “very good” health levels accounted for 7.13%. The means for smartphone usage, smartphone usage ability and smartphone usage purposes were 0.34, 4.07, and 0.61, respectively. That is, the proportion of those surveyed who use smartphones was 34.90%. Among the sample of those who use smartphones, the proportion of those who use smartphone to search for learning and health information was 61.60%. For the elderly respondents’ smartphone usage ability, from “very weak” to “strong”, the percentages obtained were 3.60, 12.30, 1.30, 38, and 44.50%, respectively.

From the perspective of the individual characteristics of the participants, male respondents accounted for 68%, female respondents accounted for 31.36%. A total of 11.80% of the participants of the study were aged between 50 and 60 years; 24.00% were between 61 and 65 years old; 25.70% were between 66 and 70 years old; 21.80% were between 71 and 75 years old; 11.90% were between 75 and 80 years old; 4.40% were 80 years old or over. A total of 3.80% of the participants of the study had a primary school education level or below; 12.50% had a junior high school level; 43.80% elderly had a senior high school or technical secondary school level; 39.10% had a university level; 0.50% had a master’s degree or above.

From the perspective of the family characteristics of the elderly, 58.80% of the participants of the study had 1-2 children; 39.30% had 3-5 children; 1.80% had more than 6 children. A total of 1% of the participants of the study rated their children’s living conditions as “poor”; 39.70% rated their children’s living conditions as “average”; 47.30% rated their children’s living conditions as “good”; 11.80% rated their children’s living conditions as “very good”. A total of 54.80% of the respondents lived with their spouses, 14.30% lived with their children, and 25.80% lived with their spouses and children.

From the socio-economic characteristics of the elderly, the mean monthly income of the elderly is 3.45, 2.53% of the elderly with incomes below 1000 yuan, 11.14% of the elderly with incomes between1,000-2000 yuan, 34.77% of the elderly with incomes between 2000 and 3000 yuan, 41.55% of the elderly with incomes between 3000 and 4000 yuan, and 9.99% of the elderly with incomes above 4000 yuan. In terms of current work with the elderly, a total of 83.30% of the elderly had retired, 11.30% were re-employed after retirement, and 2.10% had started their own businesses. In the sample, 3.70% of the elderly lived in rural areas; 6% in towns; 90.10% in counties, cities and districts. The descriptive statistics of the main variables in this paper are shown in Table 1.

As shown in Table 2, differences between the means of the health levels of elderly people who do and do not use smartphones were found. The mean health level of those who use smartphones was 3.49, and the mean health level of those who do not use smartphones was 3.16. Using the independent sample T-test in SPSS for analysis, the mean health level of the respondents who use smartphones was found to be significantly higher than that of those who do not use smartphones.

**Table 2:** Mean comparison between health levels of elderly people whether they use smartphones or not

Group	Number of samples	Mean	Standard deviation of health level
Using smartphone	1045	3.49a	0.755
Not using smartphone	1997	3.16b	0.811

Note: Different letters in the superscript (volume 3) indicate significance at 1% between the two mean values

As shown in Table 3, it was found that the means of the health levels of the elderly groups with different smartphone usage abilities were different. The physical level means of the elderly participants with weak-to-strong smartphone usage ability were 1, 3.40, 3.57, 3.49, and 3.53, respectively. We evaluated the elderly smartphone usage ability by assessing whether they could use them for online shopping, transportation, and medical care, and other functions, and their usage frequency. The results showed a polarization of the elderly smartphone usage ability, resulting in the lowest number of seniors with moderate smartphone use ability (14), and they had the highest health level (3.57). The small number resulted in their health level being more discrete. Therefore, there is not much significance in the mean value. Through one-way analysis of variance (ANOVA) in SPSS, we compared the means of one group with the other four groups and eventually achieved a comparison of means between every two groups. It was found that the average health level of the participants with a strong smartphone usage ability was significantly higher than that of those with a weak smartphone usage ability.

**Table 3:** Mean comparison between health levels of elderly people with different smartphone usage ability

Group	Number of samples	Mean	Standard deviation of health level
Very poor	38	1a	0.000
Poor	129	3.40ac	0.775
Average	14	3.57ab	0.852
Strong	398	3.49bc	0.744
Very strong	466	3.53b	0.762

Note: Different letters in the superscript (volume 3) indicate significance at 5% between the two mean values

As shown in Table 4, it was found that the means of the health levels of the elderly groups with different smartphone usage purposes were different. The mean health levels of the participants who use their smartphones to learn and search for health information was 3.55, and the mean health level of those who do not use their smartphones to learn and search for health information was 3.39. Through the independent sample T-test in SPSS, the mean health level of the elderly participants who use their smartphones to learn and search for health information was found to be significantly higher than that of those who do not use their smartphones to learn and search for health information.

**Table 4:** Mean comparison between health levels of elderly people with different smartphone usage purposes

Group	Number of samples	Mean	Standard deviation of health level
Learning and searching health information	644	3.55a	0.766
Not Learning and searching health information	401	3.39b	0.728

Note: Different letters in the superscript (volume 3) indicate significance at 1% between the two mean values

### Estimated results

This paper used three regression models to analyze the effect of smartphones on the self-rated health levels of the elderly. In Table 5, Model 1 was the analysis of the impact of smartphone usage on the self-rated health levels of the elderly. Model 2 was the analysis of the impact of smartphone usage ability on the self-rated health levels of the elderly. Model 3 was the analysis of the impact of smartphone usage purposes on the self-rated health levels of the elderly.

**Table 5:** Effect of smartphones on self-rated health level of the elderly

Variable	Self-rated health level of the elderly		
	Model 1	Model 2	Model 3
*Usage*	0.344*** (0.075)		
*Ability*		0.153** (0.053)	
*Purpose*			0.241** (0.135)
*Gender*	0.169* (0.084)	−0.041 (0.146)	− 0.037 (0.144)
*Age*	−0.365*** (0.032)	− 0.253*** (0.067)	− 0.253*** (0.065)
*Edu*	0.101** (0.055)	0.282*** (0.103)	−0.308** (0.106)
*Spouse*	1.982*** (0.742)	3.278** (1.648)	3.291** (1.564)
*Children*	1.854** (0.744)	3.079* (1.652)	3.123** (1.569)
*SC*	2.025*** (0.743)	3.427** (1.647)	3.453** (1.563)
*Conditions*	0.841*** (0.064)	0.841*** (0.113)	0.829*** (0.112)
*Number*	0.034 (0.078)	−0.171 (0.158)	−0.162 (0.158)
*Income*	0.134*** (0.051)	0.194** (0.090)	0.189** (0.090)
*Retirement*	−0.047 (0.219)	−0.401 (0.820)	− 0.443 (0.825)
*Employment*	0.419 (0.250)	0.069 (0.835)	−0.003 (0.840)
*Business*	0.069 (0.305)	0.034 (0.893)	0.032 (0.897)
*Residence*	0.003 (0.044)	−0.021 (0.077)	0.034 (0.080)
Wald chi2	409.37***	127.69***	129.53***
Pseudo R2	0.076	0.069	0.070
Log pseudolikelihood	− 3347.7337	− 1065.4205	− 1063.9284
N	3042	1045	1045

In Table 5, the three independent variables (smartphone usage, smartphone usage ability and smartphone usage purposes) all had a significant positive effect on the health level of the elderly. In Model 1, smartphone usage had a significant positive impact on the self-rated health levels of the participants; this was significant at the 1% level. The model showed that the use of smartphones has improved the self-rated health of the participants. The possible reason was that the popularization of the Internet, and especially smartphones, has expanded the intergenerational interaction mode between the elderly and their children [37]. Further, smartphones have also increased the lives and entertainment of the elderly. This is consistent with the conclusion of Ramón-Jerónimo, M.A [38].

In Model 2, the ability to use smartphones had a significant positive impact on the self-rated health levels of the participants; this was significant at the 5% level. The model showed that the participants with a stronger ability to use smartphones had higher self-rated health. The possible reason was that with the popularization of the Internet, the concept of “Internet +” has also been developed rapidly in China. It is possible to make appointments for registration, ticket purchase [39], study, shopping and entertainment through the Internet [40]. Correspondingly, people need to acquire the ability to operate more complex technological products. Therefore, the elderly with stronger smartphone usage ability are more capable of using the Internet to create convenience for themselves. This is consistent with the conclusion of Crabb, R.M [39].

In Model 3, the use of smartphones by the participants to learn or search for health information had a significant positive impact on their self-rated health levels; this was significant at the 5% level. The model showed that with the penetration of the Internet in various industries, the elderly have learned to use smartphones to search for health information to improve their health knowledge literacy levels. This is consistent with the conclusion of Crabb, R.M [39].

In Table 5, the individual characteristics, family characteristics and socio-economic characteristics of the elderly all had a certain impact on their self-rated health levels.

The age of the participants significantly negatively affected their self-rated health levels; this was significant at the 1% level. As age increases, the learning ability and cognitive behavior of the elderly are degraded, and their health status becomes worse [26]. The income, education levels, and living conditions of the elderly, and the living conditions of their children, significantly positively affected the self-rated health levels of the elderly; these were significant at the 1% level. An increase in the income of an elderly person enables them to obtain better material security. The elderly live with other people, which improves their mental and self-rated health. The living conditions of the children of the elderly also promotes the health of the elderly. The higher the education level of an elderly person, the stronger their ability to accept new things [40]. The residence, current work and number of children of the elderly had no significant effect on their self-rated health levels.

### Robustness test

As for the index selection of the Internet use of the elderly, the questionnaire included “whether to use the computer to surf the Internet”. It also reflected the daily Internet usage of the elderly. Therefore, this paper selected computer Internet usage as the replacement variable for the robustness test. In Table 6, computer usage, computer usage ability, and learning or searching for health information online all had a significant positive impact on the self-rated health levels of the elderly. They were significant at the levels of 5, 5 and 1%, respectively. This indicates that computer usage has improved the self-rated health level of the participants. The conclusion after replacing the variables was consistent with the results of smartphone usage, which shows that the research results had robustness.

**Table 6:** Robustness test

Variable	Self-rated health level of the elderly		
	Model 1	Model 2	Model 3
*Computer usage*	0.589** (0.071)		
*Computer usage ability*		0.107** (0.043)	
*Computer usage purpose*			0.368*** (0.104)
Control variable	Controlled	Controlled	Controlled
Wald chi2	411.48***	252.08***	252.32***
Pseudo R2	0.074	0.080	0.078
Log pseudo likelihood	− 3354.1523	− 1869.1175	− 1872.3037
*N*	3042	1766	1766

### The net effect of the use of smartphones on the self-rated health levels of the elderly

Considering that there may be endogenous problems in the model due to “self-selection bias”, and in order to test whether the impact effect of smartphone use on the self-rated health levels of the elderly was consistent, this paper used the propensity score matching (PSM) method to re-estimate the relationship between Internet usage and the health of the elderly. Three methods, k-nearest neighbor matching, kernel matching and radius matching, were used. Through the balanced matching test, it was found that the matched samples had a good balance.

In Table 7, the results showed that the sample after PSM matching passed the balance test, and the selectivity bias of the sample was largely eliminated [26]. The standard deviation of all variables after matching dropped to less than 10%. The t-value differences of each variable before and after the matching gradually decreased. After the matching, the *p*-value of the t-test of each variable was mostly greater than 0.1. The matched Pseudo-R2 value (0.118 → 0.004), LR chi2 (461.11 → 10.46), MeanBias (21.9% → 2.6%), and MedBias (20.8% → 2.6%) were significantly lower; while the joint significance test for the control variables also became less significant. Additionally, the B value was 14.1, which was less than 25%. What’s more, the R value was 1.37, which is between 0.5 and 2. All the results indicated that the sample matching was successful, and there was no difference in the distribution of control variables between older adults who used the Internet and those who did not after matching.

**Table 7:** Sample balance test

	Variable	Experience group	Control group	Standard deviation (%)	Deviation reduction(%)	T-value	*P*-value
*Gender*	Unmatched	0.667	0.691	−5.0		−1.28	0.199
	Matched	0.667	0.634	7.2	−44.6	1.59	0.112
*Age*	Unmatched	2.666	3.326	−51.4		−13.00	0.000
	Matched	2.666	2.692	−2.1	95.9	−0.50	0.616
*Edu*	Unmatched	3.401	3.084	41.4		10.28	0.000
	Matched	3.401	3.431	−3.9	90.6	−1.00	0.316
*Living situation*							
Spouse	Unmatched	0.552	0.547	1.0		0.25	0.799
	Matched	0.552	0.550	0.4	60.2	0.09	0.930
*Children*	Unmatched	0.144	0.143	0.2		0.06	0.953
	Matched	0.144	0.143	0.3	−23.0	0.06	0.950
*SC*	Unmatched	0.266	0.254	2.9		0.13	0.899
	Matched	0.266	0.270	− 0.9	69.5	− 0.20	0.843
*Conditions*	Unmatched	2.879	2.6095	39.7		10.26	0.000
	Matched	2.879	2.840	5.7	85.7	1.27	0.203
*Number*	Unmatched	2.305	2.493	−36.7		−9.23	0.000
	Matched	2.305	2.279	5.0	86.3	1.23	0.22
*Income*	Unmatched	3.637	3.351	32.2		8.15	0.000
	Matched	3.637	3.694	−6.5	79.9	−1.52	0.129
*Current work*							
*Retirement*	Unmatched	0.786	0.858	−19.0		−5.10	0.000
	Matched	0.789	0.817	−7.3	61.4	−1.60	0.110
*Employment*	Unmatched	0.180	0.078	31.0		8.59	0.000
	Matched	0.177	0.160	5.2	83.2	1.05	0.293
*Business*	Unmatched	0.026	0.018	5.9		1.60	0.110
	Matched	0.026	0.020	4.5	23.2	1.01	0.312
*Residence*	Unmatched	3.516	3.313	21.6		5.41	0.000
	Matched	3.516	3.601	−9.1	58.0	−2.18	0.029
	Pseudo R2	LR chi2	*p* > chi2	MeanBias (%)	MedBia(%)	B	R
Unmatched	0.118	461.11	0.000	21.9	20.8	84.9*	0.84
Matched	0.004	10.46	0.656	2.6	2.6	14.1	1.37

Note: In radius matching, the radius is set to 0.05; in kernel matching, the kernel function and broadband use default values; k-nearest neighbor matching selects k = 1

Table 8 showed the average treatment effect on the treated (ATT) of the self-rated health levels of the elderly participants who use smartphones. Three matching methods, k-nearest neighbor matching, kernel matching and radius matching, were used to measure the robustness of the results by comparing the results of the three methods to a certain extent. The specific performance is as follows: The results of the three matching methods of k-nearest neighbor matching, kernel matching and radius matching were 13.26, 15.33 and 14.80%, respectively. The results demonstrated that the elderly who used the Internet were about 14.46% (the mean of 13.26, 15.33 and 14.80%) healthier than those who did not use the Internet. The results of the three matching methods were similar, meaning that whichever matching method was used led to the consistent conclusion that Internet use can improve the health of the elderly. Also it can be seen that the results after the propensity matching of scores were robust.

**Table 8:** Results of propensity score matching method (PSM)

Analysis method	ATT	Standard error	T-value
Radius Matching	0.132	0.035	3.74
Kernel Matching	0.153	0.034	4.48
K-Nearest Neighbor Matching	0.148	0.051	2.86

## Discussion

### Study strengths

The contribution of this research was mainly reflected in two aspects.

- Firstly, unlike previous studies that only focused on whether the elderly use smartphones, this study found more in-depth information and conclusions in multiple categories. We explored the effect of smartphones on the self-rated health levels of the elderly from three aspects: smartphone usage, smartphone usage ability, and smartphone usage purposes. The study found that these all improved the self-rated health of the elderly. In addition, we also used computer usage as a replacement variable to test the robustness of the research results. After replacing the variables, the conclusion was consistent with the results regarding smartphone usage, which shows good robustness.
- Secondly, this study not only used the multivariate ordered logistic model to explore the effect of smartphone usage on the self-rated health levels of the elderly, but also further used the PSM method to explore the net effect of smartphone usage on the elderly’s self-rated health levels. We respectively used three matching methods, k-nearest neighbor matching, kernel matching and radius matching, for measurement, and obtained more robust results.

### Study limitations

Although this research is helpful in promoting the integration of the elderly into the information society, there are still the following shortcomings, which need to be further studied. First, this article just focuses on the self-rated health levels of the elderly. In the discussion of active aging, we should also pay attention to the mental health of the elderly. Second, the concept of “Internet+pension” is in the initial stage of advancement, and the development of the Internet itself constitutes a dynamic process of change. In this process, the impact of Internet factors on the health of the elderly may also change accordingly. Future research should consider the impact of dynamic changes on the use of the Internet by the elderly.

## Conclusions

This paper aimed to explored the impact of smartphone usage on the self-rated health of the elderly. It used a questionnaire survey on the use of Internet technology by elderly people over 55 years of age from a sample survey of Jiangxi Province in 2018. Further, the study analyzed the impact of smartphone usage ability and smartphone usage purposes on the level of self-rated health of the elderly. It mainly used the multivariate ordered logistic model and the propensity score matching (PSM) method. The main conclusions were as follows:

- First, the investigation showed that 34% of the participants use smartphones, and 61% of them use smartphones for learning and searching for health knowledge. The mean value of the smartphone usage ability of the participants was 4.01, which was at a high level. We compared the mean values between the health of elderly people with different levels of smartphone usage, smartphone usage ability, and smartphone usage purposes. It was found that the health levels of the participants who used smartphones was higher than those who did not use smartphones. The elderly with high smartphone use ability had higher health levels than those with low smartphone use ability. The elderly who used smartphones to learn and search for health knowledge had higher health levels than those who did not use smartphones to learn and search for health knowledge.
- Second, this paper analyzed the effects of smartphones on the health of the elderly using a multivariate ordered logistic model, including smartphone usage, smartphone usage ability, and smartphone usage purposes. The results showed that smartphone usage, smartphone usage ability, and smartphone usage purposes all had a significant positive effect on the self-rated health of the elderly, with 1, 5, and 5% effects, respectively. The PSM model was further used to calculate the net effect, including k-nearest neighbor matching, kernel matching and radius matching, and the results of the three matching methods demonstrated that the net effect of smartphone usage on the self-rated health levels of the elderly were 13.26, 15.33 and 14.80%, respectively.

## Data Availability

The datasets generated and/or analyzed during the current study are not publicly available because some results are still being analyzed but are available from the corresponding author on reasonable request.

## Abbreviations

PSM: Propensity score matching
CNNIC: China Internet Network Information Center
HIL: Health information literacy
